# Pathogenic Factor Analysis of Shoulder Periarthritis and Design of Virtual Reality Exercise Intervention System

**DOI:** 10.1155/abb/6543337

**Published:** 2024-12-19

**Authors:** Yucheng Tian, Dan Qiu, Renjie Song, Xue Cheng, Feiyu Chen, Dongqing Sun, Yiduo Zhou, Shaomin Cai, Zhaowei Wang, Weijia Zhang

**Affiliations:** ^1^School of Mathematical and Information Science, Shaoxing University, Shaoxing 312000, China; ^2^School of Medicine, Shaoxing University, Shaoxing, China; ^3^Key Laboratory of Artificial Intelligence Applications, Shaoxing University, Shaoxing, China; ^4^Department of AOP Physics, University of Oxford, Oxford, UK

## Abstract

Shoulder periarthritis, a prevalent musculoskeletal disorder, causes significant pain and functional impairment, severely affecting patients' quality of life. With the increasing incidence of shoulder periarthritis linked to modern lifestyle changes, effective prevention and treatment strategies remain elusive. This study explores two areas: first, identifying risk factors for shoulder periarthritis through Mendelian randomization (MR) analysis, and second, designing a motion intervention system incorporating MediaPipe and virtual reality (VR) technology. The MR analysis revealed positive causal relationships between shoulder periarthritis and body mass index (BMI), cigarettes per day, insomnia, and sedentary behavior, while higher educational attainment was inversely associated with the condition. Based on these findings, we developed a “VR Harvest” exercise system aimed at alleviating shoulder stiffness due to prolonged sitting and reducing shoulder pain. By adjusting task difficulty to increase physical activity, the system also facilitates BMI reduction, potentially lowering the risk of shoulder periarthritis. The experimental results indicate that the “VR Harvest” therapy effectively alleviates pain and, compared to traditional exercise therapy, offers greater enjoyment, thereby enhancing participant engagement. Moreover, participants across different BMI categories experienced reductions in BMI after completing the intervention. This study offers a novel approach for shoulder periarthritis prevention and treatment, leveraging VR technology to improve both symptom relief and underlying risk factors.

## 1. Introduction

Shoulder periarthritis is a common condition characterized by shoulder pain and restricted movement, drawing significant attention due to its high incidence and profound impact on patients' quality of life. Epidemiological studies indicate that the prevalence of shoulder periarthritis ranges from approximately 2% to 5%, with a slightly higher occurrence in women than in men [1]. The prevalence of shoulder periarthritis increases significantly among individuals around the age of 50, leading to the condition being commonly referred to as “50 shoulder” [2]. The rising incidence of shoulder periarthritis poses a significant health and economic burden on both individuals and society. While primarily affecting middle-aged and older adults, it also impacts younger populations, particularly athletes and laborers, severely hindering their work, sports activities, and daily life.

In recent years, shifts in lifestyle and the increasing prevalence of obesity have garnered widespread attention regarding the relationship between obesity and various diseases. Body mass index (BMI), a commonly used indicator of obesity, has not yet been fully established as a causal factor for shoulder periarthritis. Several studies suggest that obesity may elevate the risk of developing shoulder periarthritis through various mechanisms. For instance, excess body weight can impose additional stress on the shoulder joint, leading to cartilage wear and exacerbating inflammatory responses [3]. Furthermore, obesity is often associated with metabolic disorders such as insulin resistance and chronic inflammation, which may also contribute to the development of shoulder periarthritis [4].

However, definitively establishing causal relationships between various factors and shoulder periarthritis is challenging. Traditional observational studies are often susceptible to confounding factors and reverse causation, which can lead to inaccurate conclusions. Therefore, more advanced research methods, such as Mendelian randomization (MR), are essential. MR employs genetic variants as instrumental variables (IVs); because these genetic variants are randomly assigned during gamete formation and are not influenced by environmental or lifestyle factors, this approach effectively mitigates the impact of confounding variables, allowing for a more reliable inference of causal relationships.

In terms of treatment, traditional methods for managing shoulder periarthritis include physical therapy, medication, and surgical interventions; however, these approaches have certain limitations regarding therapeutic efficacy and patient compliance. With the rapid advancement of virtual reality (VR) technology and deep learning, MediaPipe offers an advanced posture estimation framework with robust motion analysis capabilities, enabling accurate tracking and analysis of patients' postures and movements. The immersive and interactive characteristics of VR technology demonstrate significant potential for application in the medical field. In this study, we combined MediaPipe with VR to design an exercise intervention system. MediaPipe, serving as a tool for human posture detection and motion recognition, can seamlessly integrate with VR technology to monitor and evaluate the shoulder movements of subjects in real time during exercise. This integration allows for a more precise assessment of the effects of exercise therapy, providing a more scientific basis for the treatment of shoulder periarthritis.

VR technology also demonstrates significant potential in the realm of medical rehabilitation. In the field of stroke rehabilitation, researchers have innovatively developed a clinical neurorehabilitation assessment and training model centered on VR, integrating electromyography, electroencephalogram recognition and analysis, and motion capture technologies based on the principle of neuroplasticity. Following this training, patients have been able to regain a certain degree of control over the affected ankle, resulting in restored motor function. In other rehabilitation fields, VR technology has also found various applications. Matthie et al. [5] used VR technology as a nonpharmacological therapy for chronic pain, with a randomized controlled trial demonstrating its effectiveness in alleviating patients' pain 1 month posttreatment. Similarly, Lorenz et al. [6] enhanced visuospatial dysfunction in patients by enabling them to engage in repeated navigation and positioning training within a virtual supermarket.

This study aims to explore the causal relationships between lifestyle habits, physical conditions, educational background, and shoulder periarthritis by utilizing MR analysis. Additionally, it seeks to assess the effectiveness and feasibility of VR technology in the treatment of shoulder periarthritis. Through this research, we hope to provide new theoretical insights and practical guidance for the prevention and treatment of shoulder periarthritis, ultimately offering patients more effective treatment options to alleviate their pain and improve their quality of life.

## 2. Pathogenic Factor Analysis of Shoulder Periarthritis

### 2.1. Research Methods

MR is an epidemiological method that uses genetic variants as IVs to explore causal relationships. The method is based on Mendel's laws of inheritance, which assume that gene distribution is random and that genetic variation is independent of environmental factors within a population. MR analysis resembles a randomized controlled trial [7], where single nucleotide polymorphisms (SNPs) serve as IVs to infer causal relationships between exposures and outcomes [8]. By utilizing genetic variants as IVs, MR analysis minimizes bias caused by confounding variables and effectively addresses the issue of reverse causality. This method is frequently used to infer various clinical causal relationships.

In our study, we used data from genome-wide association studies (GWAS) and conducted a two-sample MR analysis to investigate the causal relationships between BMI, cigarettes per day, sedentary behavior, insomnia, educational attainment, and shoulder periarthritis.

### 2.2. Data Sources

The datasets for BMI (ieu-b-40) and educational attainment (ieu-b-6134) were obtained from the IEU OpenGWAS project website. The data about cigarettes per day, sedentary behavior, and insomnia were obtained from the GWAS catalog. The summarized statistical data for shoulder periarthritis were obtained from the FinnGen R8 dataset. The GWAS data for leisure-time television viewing behavior were used as a substitute for sedentary behavior.

### 2.3. Data Processing

MR analysis is a powerful tool in epidemiological studies. In this study, we primarily employed inverse variance weighted (IVW) [9], MR-Egger regression [10], weighted median (WM) [11], and weighted mode for MR analysis.

In this study, the genetic instruments used for MR analysis must satisfy three core assumptions: the relevance assumption [12], the independence assumption [13], and the exclusivity assumption [14]. SNPs that achieved genome-wide significance (*p*  < 5 × 10^−8^) were selected as IVs for MR analysis. The linkage disequilibrium coefficient (*r*^2^) was set to 0.001, and the distance threshold was set at 10,000 kilobases (kb) to ensure the independence of each SNP and to exclude the influence of gene pleiotropy on the results [15]. The *F* statistic was calculated using the following formula to evaluate the strength of the IVs, where *N* is the sample size, *k* is the number of SNPs, and *R*^2^ is the proportion of variation explained by SNPs in the exposure database. The SNPs selected in this study were all based on the strong correlation standard of *F* > 10 [16].

(1) $$\begin{array}{c} F=\frac{R^{2}\left(N-k-1\right)}{k\left(1-R^{2}\right)}. \end{array}$$

Table 1 shows the effect allele frequency and its corresponding *p* values for the IVs and exposure factors. Given the space limitations of this article, only selected SNPs are presented.

### 2.4. Results of Analysis

**Figure 1:**
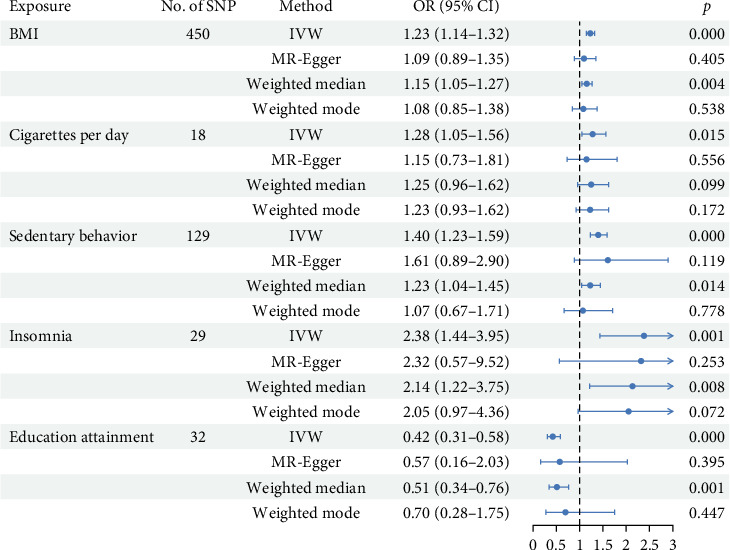
The forest plots for Mendelian randomization (MR) causal estimates of shoulder periarthritis. An odds ratio (OR) value greater than 1 indicates an increased risk, while an OR value less than 1 indicates a decreased risk.

A total of 450, 18, 129, 29, and 32 SNPs were used as IVs for BMI, cigarettes per day, sedentary behavior, insomnia, and educational attainment, respectively. The results obtained using IVW are shown in Figure 1 and Table 2.

BMI: For each increase in BMI by one standard deviation (1-SD), the risk of developing shoulder periarthritis increased by 23% (odds ratio (OR): 1.23, 95% confidence interval (CI): 1.14–1.32, and *p* = 2.61 × 10^−8^).

Cigarettes per day: Based on 18 SNPs, each 1-SD increase in cigarettes per day was associated with a 28% increase in the risk of developing shoulder periarthritis (OR: 1.28, 95% CI: 1.05–1.56, and *p* = 0.01).

Insomnia: Utilizing 29 SNPs, each 1-SD increase in insomnia was associated with approximately a 2.4-fold increase in the risk of developing shoulder periarthritis (OR: 2.38, 95% CI: 1.44–3.95, and *p* = 0.0007).

Educational attainment: Based on 32 SNPs, an increase in educational attainment was found to have a protective effect against the risk of developing shoulder periarthritis (OR: 0.42, 95% CI: 0.31–0.58, and *p* = 1.89 × 10^−7^).

Sedentary behavior: Using 129 SNPs, each 1-SD increase in sedentary behavior was associated with a 40% increase in the risk of developing shoulder periarthritis (OR: 1.40, 95% CI: 1.23–1.59, and *p* = 2.59 × 10^−7^).

This study investigated the stability of causal associations between various exposure factors and shoulder periarthritis. We employed multiple statistical models, including IVW, MR-Egger regression, WM, and weighted mode, for a comprehensive evaluation (Table 2).

Taking BMI as an example, both IVW and WM analyses indicated a strong causal association between increased BMI and the risk of shoulder periarthritis (*p*  < 0.01). However, the weighted mode and MR-Egger regression did not reveal significant causal associations (*p*  > 0.05). Similarly, while IVW and WM analyses indicated an increased risk of shoulder periarthritis associated with insomnia (*p*  < 0.05), MR-Egger regression and weighted mode did not show significant results (*p*  > 0.05).

For cigarettes per day, IVW indicated a significant association with the risk of shoulder periarthritis (*p* = 0.015). In contrast, MR-Egger regression, WM, and weighted mode did not significantly affect this association (*p*  > 0.05).

Regarding educational attainment, both IVW and WM analyses indicated that higher educational attainment is associated with a reduced risk of shoulder periarthritis (*p*  < 0.05). However, neither MR-Egger regression nor the weighted mode showed significant effects on this association (*p*  > 0.05).

In contrast to MR-Egger regression and weighted mode, both IVW and WM analyses indicated a significant causal association between sedentary behavior and the risk of shoulder periarthritis (*p*  < 0.05).

### 2.5. Horizontal Pleiotropy Analysis

**Figure 2:**
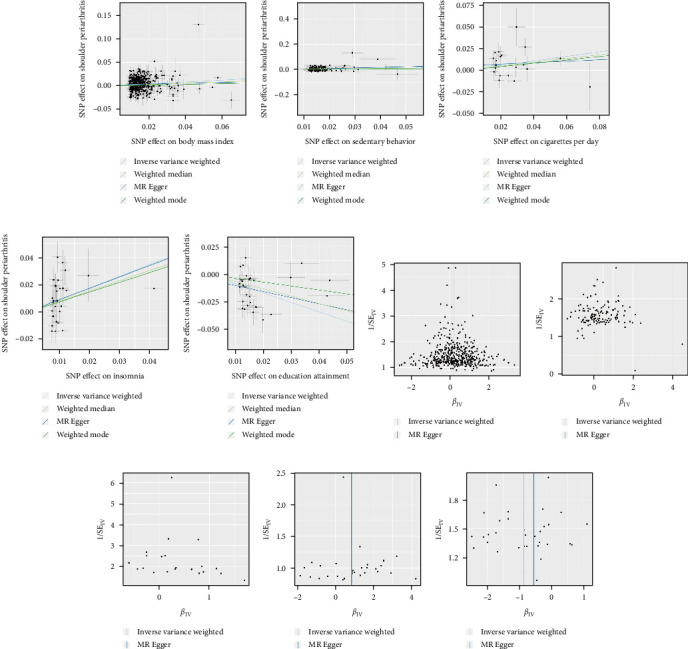
Scatter plots and funnel plots of each exposure variable and shoulder periarthritis. (a) Scatter plot of body mass index (BMI) and shoulder periarthritis. (b) Scatter plot of sedentary behavior and shoulder periarthritis. (c) Scatter plot of cigarettes per day and shoulder periarthritis. (d) Scatter plot of insomnia and shoulder periarthritis. (e) Scatter plot of educational attainment and shoulder periarthritis. (f) Funnel plot of BMI and shoulder periarthritis. (g) Funnel plot of sedentary behavior and shoulder periarthritis. (h) Funnel plot of cigarettes per day and shoulder periarthritis. (i) Funnel plot of insomnia and shoulder periarthritis. (j) Funnel plot of educational attainment and shoulder periarthritis.

To further examine the potential influence of horizontal pleiotropy, scatter plots and funnel plots, as shown in Figure 2, were utilized to visually display the associations between the exposure variables (cigarettes per day, sedentary behavior, BMI, and educational attainment) and shoulder periarthritis. According to Table 3, heterogeneity was observed in the exposure variables of educational attainment, insomnia, BMI, and sedentary behavior; however, the IVW model was primarily employed. No heterogeneity was detected for cigarettes per day. Additionally, the *p*-values from the MR-Egger regression intercept tests were all greater than 0.05, indicating no evidence of horizontal pleiotropy for these exposure variables.

### 2.6. Results of MR Analysis

Based on the two-sample MR analysis, this study identified positive causal relationships between BMI, cigarettes per day, insomnia, sedentary behavior, and shoulder periarthritis. These findings indicate that increases in these factors elevate the risk of developing shoulder periarthritis. Conversely, a negative causal relationship was observed between educational attainment and shoulder periarthritis, with higher levels of education associated with a lower risk.

## 3. Design and Implementation of a VR Exercise Intervention System

The MR analysis indicates that patients with a higher BMI are more prone to developing shoulder periarthritis compared to those with a lower BMI, and that sedentary behavior increases the risk of this condition. Based on these findings, we propose the design of an exercise intervention system aimed at reducing BMI and strengthening shoulder muscles to alleviate stiffness caused by prolonged inactivity. This system could serve both to prevent shoulder periarthritis and to relieve shoulder pain in those already suffering from the condition.

Traditional rehabilitation exercises often need to be conducted in specialized facilities under the supervision of a physician, which poses significant limitations. Additionally, the process can be monotonous and lacks engagement. To address these challenges, this study employs MediaPipe and VR technology, combined with the Unity3D game engine, to integrate rehabilitation exercises into a gaming environment. The result is a VR exercise intervention system that is location independent, easy to use, and highly engaging.

### 3.1. Components of the VR Exercise Intervention System

**Figure 3:**
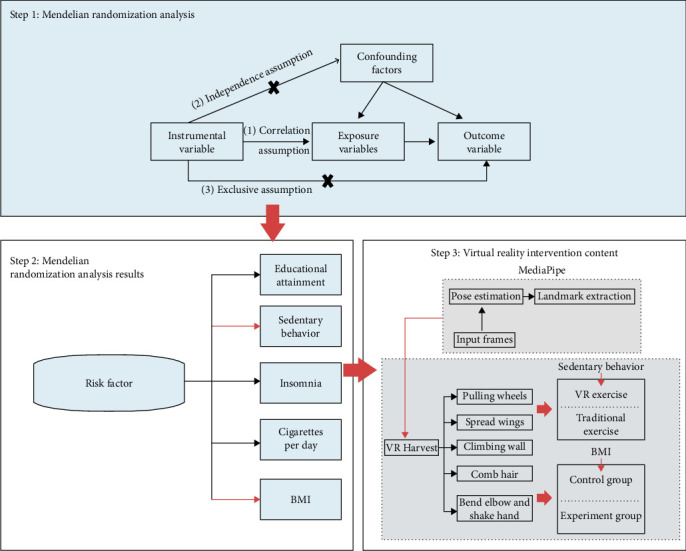
The training activities of the virtual reality (VR) exercise intervention system.

The VR exercise intervention system includes two exercise intervention plans. One plan combines MediaPipe and VR to immerse participants in a virtual environment where they engage in “VR Harvest,” directly targeting shoulder muscles to treat shoulder periarthritis. The other plan adjusts the intensity of shoulder muscle exercises, increasing the workout volume during the picking activity to reduce BMI, thereby indirectly lowering the risk of shoulder periarthritis.  Figure 3 illustrates the training content of the VR exercise intervention system.

### 3.2. Application of MediaPipe in the System

MediaPipe is a cross-platform real-time pose tracking framework that uses a camera to detect and track body posture in real time. In this system, MediaPipe is used to capture user movements, enabling interactive support for VR exercise interventions. As users engage in exercises within the VR environment, MediaPipe performs real-time analysis of the input image or video stream, using convolutional neural networks (CNNs) based on deep learning to extract key human body points such as the head, shoulders, elbows, wrists, and knees. By analyzing these key points, the system can accurately track the user's posture and movement, matching this information to preset exercise protocols.

The BlazePose module within MediaPipe is specifically employed to detect and track joint positions, providing feedback during exercises. As users move, MediaPipe analyzes movement angles, range of motion, and speed, generating real-time assessments and posture feedback to ensure the accuracy and safety of each exercise. This not only enhances the interactivity of training, but also offers personalized exercise guidance to the user.

By integrating MediaPipe with VR, the system can accurately evaluate the user's posture during exercise and respond accordingly within the virtual environment. For instance, in a shoulder exercise intervention, the system assesses whether the user's shoulder and elbow movements meet therapeutic requirements, based on the position of these key points. If adjustments are needed, the system provides corrective feedback. This feedback mechanism helps enhance the effectiveness of exercise therapy and improves the precision and safety of the training.

### 3.3. Design of VR Exercise Therapy for Shoulder Periarthritis

In today's society, an increasing number of workers are required to sit at their desks for extended periods. This work style often results in the muscles around the shoulders remaining relatively static for long durations. Prolonged inactivity can lead to muscle tension and stiffness, limiting shoulder mobility. Additionally, extended periods of sitting can prevent the shoulder muscles from receiving sufficient exercise, causing a gradual decline in muscle strength. Long hours of sitting may also contribute to poor posture, such as slouching or forward-rounded shoulders. These improper postures increase stress on the shoulder joints and muscles, potentially leading to conditions like shoulder periarthritis.

#### 3.3.1. Experimental Analysis of the Impact of VR Exercise Therapy on Shoulder Pain

This study developed a shoulder movement intervention protocol incorporating MediaPipe and VR technologies to evaluate their efficacy in alleviating pain and enhancing shoulder joint functionality in individuals with shoulder periarthritis. The experimental group underwent VR-based exercise therapy, with pain levels assessed using the visual analog scale (VAS), where scores range from 0 (no pain) to 10 (severe pain).

**Figure 4:**
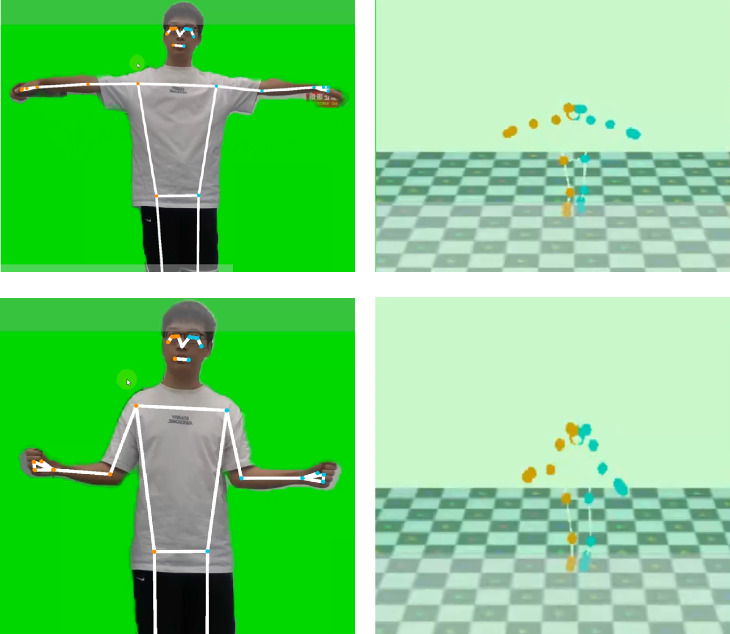
Virtual reality (VR) Harvest part action display. (a) Training action images generated by MediaPipe. (b) Generating human skeletal structure based on key point information extracted by MediaPipe.

During the intervention, participants practiced shoulder exercises within a “VR Harvest” scenario. A series of shoulder rehabilitation movements were executed, including wheel pulls, wall climbs, wing spreads, combing motions, and elbow flexion and arm swings, as outlined in Table 4. Each exercise incorporated virtual apple-picking tasks to increase shoulder muscle flexibility and joint mobility. As illustrated in Figure 4, the system leveraged MediaPipe to capture participants' key postural points during the “VR Harvest” exercises, providing a real-time analysis of shoulder movement that enhanced the evaluation of VR exercise therapy's impact on shoulder periarthritis symptoms.

The pulley pull exercise primarily targets the anterior and middle deltoids, enhancing shoulder joint mobility and alleviating stiffness and pain associated with prolonged sitting.

The wall climb exercise strengthens the deltoids and biceps, effectively increasing the range of motion in the shoulder, particularly in upward extension. This exercise can alleviate stiffness and restricted movement in patients with shoulder periarthritis.

The wing stretch corrects muscle imbalances caused by poor posture, relieving shoulder muscle tension and reducing pain and discomfort. The abduction and lifting movements enhance shoulder joint mobility, helping improve shoulder function in patients with shoulder periarthritis.

The head brushing exercise focuses on the deltoids, rotator cuff muscles, and upper arm muscles, improving flexibility and the range of motion in the shoulder. Repeated movements help relieve muscle tension while also enhancing shoulder strength and coordination to promote recovery.

Finally, the elbow flexing and arm swinging exercise targets the posterior deltoid and rotator cuff muscles, strengthening the rotator cuff and improving shoulder joint stability. This exercise can increase the external rotation range of motion and alleviate common mobility issues in patients with shoulder periarthritis.

The training of the experimental group uses VR devices and MediaPipe technology to track the subjects' shoulder movements in real time. The system provides feedback and correction based on the captured movements to ensure the accuracy and effectiveness of each movement. The control group received traditional exercise therapy, using traditional rehabilitation equipment such as dumbbells and stretching bands.

**Figure 5:**
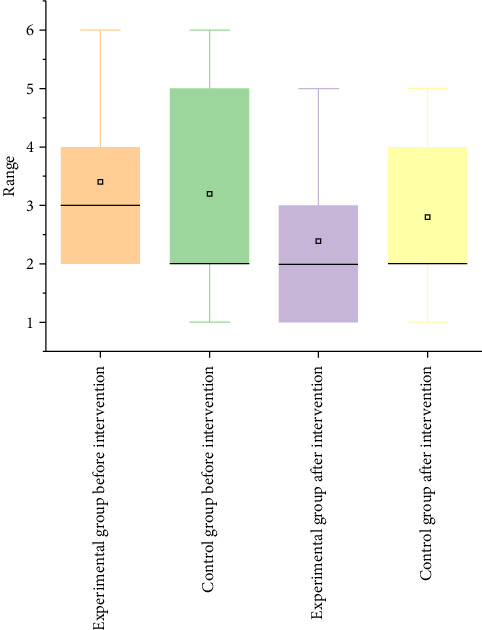
A box plot of visual analog scale (VAS) score changes in participants after a 4-week intervention, illustrating pain level variations for both the experimental group (receiving virtual reality (VR) exercise therapy) and the control group (receiving traditional exercise therapy). This comparison highlights the pre- and posttreatment VAS pain scores, showing the effectiveness of each intervention in reducing pain.

This study involved 10 patients with shoulder periarthritis, divided into an experimental group and a control group, with five patients in each group and a gender ratio of 50%. The age range of the subjects is 30–60 years old, with an average weight of 65 kg; the average height is 1.7 m. The experiment lasts for 4 weeks, with three training sessions per week, each lasting 40 min. VAS scores were evaluated before and after intervention to compare the changes in pain between the two groups of subjects, as shown in Figure 5.

#### 3.3.2. Experimental Analysis of the Impact of VR Exercise Therapy on BMI

To assess the impact of VR exercise therapy on BMI, this study recruited university students as participants for both the experimental and control groups. Both groups were matched in size and similar in physical characteristics, with each group containing five participants in a 1:1 gender ratio. Participants ranged in age from 20 to 28 years, with an average weight of 68 kg and an average height of 1.69 m. The 4-week intervention required training sessions three times per week, lasting 40 min each, to ensure comparability in results.

**Figure 6:**
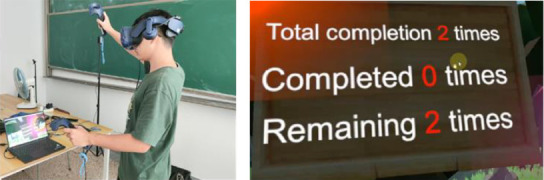
Display of shoulder periarthritis treatment through virtual reality (VR) Harvest. (a) Rehabilitation training through VR Harvest game. (b) Feedback display of VR Harvest game completion.

The experimental design involved both groups training within a virtual environment but with varying intensities: the experimental group performed high-intensity training, while the control group followed a lower-intensity regimen. The VR–based exercise focused on a simulated “VR Harvest” activity that guided participants through shoulder movements tasks within a virtual setting (Figure 6). These movements aimed to strengthen shoulder muscles, which could contribute to shoulder pain relief and improved joint mobility for individuals with shoulder conditions. Using MediaPipe technology, the system captured users' movements in real time, providing feedback on movement quality. A performance panel indicated completion accuracy, helping users execute each movement correctly and safely. This approach aims to reduce physical discomfort, correct postural imbalances, and enhance functional capabilities in daily life.

**Figure 7:**
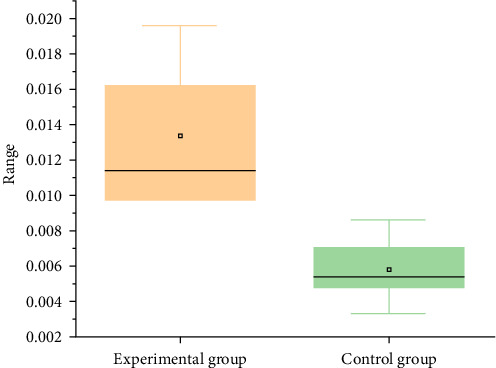
Box plot of body mass index (BMI) changes (*Δ*BMI) in the experimental and control groups. In the experimental group, the maximum *Δ*BMI is 0.01957, the minimum is 0.00978, and the median is 0.01136. In the control group, the maximum *Δ*BMI is 0.00860, the minimum is 0.00332, and the median is 0.0058.

At the end of the intervention, BMI measurements were taken for each participant pre- and postexercise. Relative BMI change (*Δ*BMI) was calculated for each individual, with results indicating the effectiveness of VR exercise therapy on BMI reduction. Detailed BMI changes for each participant are presented in Supporting Information: Table S1. Additionally, participant feedback was analyzed to examine the influence of varying training intensities on both BMI reduction and exercise enjoyment. As seen in Figure 7 and Table 5, the distribution of *Δ*BMI was slightly higher in the experimental group compared to the control group, with the experimental group demonstrating more significant BMI changes, suggesting a more pronounced effect of high-intensity VR training on BMI reduction.

  
$$\begin{array}{c} . \end{array}$$

### 3.4. Statistical Analysis

To evaluate the effects of VR exercise therapy on pain relief (VAS scores) and BMI changes in patients with shoulder periarthritis, nonparametric statistical tests were employed in this study. The data from pre- and postintervention in the experimental and control groups were analyzed using the Wilcoxon signed-rank test and the Mann–Whitney *U* test, respectively, to compare intragroup and intergroup changes.

#### 3.4.1. Wilcoxon Signed-Rank Test

The Wilcoxon signed-rank test was applied to assess intragroup comparisons of VAS scores and BMI changes before and after intervention for both the experimental and control groups, as shown in Table 6.

Results of the Wilcoxon test indicated a *p*-value of 0.025 for VAS score changes in the experimental group, which is below the significance level of 0.05. This suggests a significant reduction in pain postintervention, demonstrating the effectiveness of VR exercise therapy in alleviating pain for patients with shoulder periarthritis. For the control group, the Wilcoxon test yielded a *p*-value of 0.157, exceeding 0.05, which suggests no significant change in VAS scores.

Regarding BMI changes, the Wilcoxon test for the experimental group returned a *p*-value of 0.043, also below 0.05, indicating a downward trend in BMI postintervention. This result suggests that the increased intensity of the VR picking exercise positively impacted BMI reduction. Similarly, the control group showed a Wilcoxon *p*-value of 0.043 for BMI changes, suggesting a decreasing trend, albeit with a smaller effect than the experimental group.

#### 3.4.2. Mann–Whitney *U* Test

To evaluate differences in postintervention VAS scores and BMI changes between the experimental and control groups, a Mann–Whitney *U* test was conducted. Results are presented in Table 7.

The Mann–Whitney *U* test showed that the intergroup comparison of postintervention VAS scores yielded a *p*-value of 0.690, exceeding the significance threshold of 0.05, indicating no significant difference between the groups in terms of pain reduction. However, the intergroup comparison for *Δ*BMI yielded a *p*-value of 0.008, which is below 0.05, demonstrating a statistically significant difference between the groups in terms of BMI change.

#### 3.4.3. Statistical Analysis Summary

The results of the within-group statistical analysis indicate that the experimental group demonstrated significant changes in both VAS scores and BMI, suggesting that VR exercise therapy effectively reduces pain and improves BMI. However, between-group comparisons revealed no significant difference in postintervention VAS scores between the experimental and control groups, while a significant difference was noted in BMI. This discrepancy may be attributed to the subjective nature of VAS scores, which can be heavily influenced by individual participants, leading to the lack of statistically significant differences in this measure.

### 3.5. Experimental Summary

In the experiment evaluating the effectiveness of VR exercise therapy for alleviating shoulder pain, a 4-week rehabilitation training resulted in a reduction of the median VAS score in the experimental group from 3 to 2, as illustrated in Figure 5. This decrease indicates that the majority of participants in the experimental group experienced pain relief following treatment. In contrast, the median score in the control group remained unchanged, suggesting that the overall pain relief among control participants was minimal, with some individuals showing little to no change in their pain scores.

Furthermore, the interquartile range (IQR) of the experimental group decreased postintervention, indicating that the pain relief experienced by most participants was more consistent and that variability in the scores was reduced, reflecting a higher degree of treatment efficacy. In the control group, the IQR changes suggest that some participants experienced only minor fluctuations in pain scores, resulting in less pronounced effects compared to the experimental group. The maximum pain score in the experimental group decreased from 6 to 5, while the minimum score dropped from 2 to 1, indicating that participants experiencing moderate pain saw improvements toward lighter pain levels. In the control group, only the maximum score decreased, and overall changes were less significant than in the experimental group.

Overall, the VAS scores in the experimental group showed marked improvement, shifting from moderate to mild pain levels. Changes in median, IQR, and maximum and minimum values reflected a trend of pain reduction, while improvements in the control group were limited, with no change in median scores, a slight decrease in maximum scores, and a relatively large IQR, indicating less significant pain improvement and greater variability among participants.

In the experiment assessing the impact of VR exercise therapy on BMI reduction, results after 4 weeks, as shown in Figure 7, indicated that the *Δ*BMI in the experimental group ranged from 0.00978 to 0.01957, while the control group's *Δ*BMI ranged from 0.00332 to 0.00860. The higher training intensity in the experimental group led to increased energy expenditure and metabolic rates, resulting in a more significant decrease in BMI. Conversely, the low-intensity training in the control group had a relatively minor effect on BMI due to lower energy expenditure and metabolic rates, resulting in limited weight loss. This comparison highlights that high-intensity training significantly reduces BMI among participants.

## 4. Discussion and Conclusion

With the rapid development of sports rehabilitation medicine, sports rehabilitation training has emerged as an effective method for preventing and treating shoulder periarthritis. Rehabilitation training for patients with shoulder periarthritis essentially falls under the category of active sports rehabilitation. By employing targeted and scientific rehabilitation techniques, it is possible to improve shoulder joint function. This study, through MR analysis, concluded that there are causal relationships between BMI, cigarettes per day, educational attainment, insomnia, sedentary behavior, and shoulder periarthritis. Specifically, BMI, cigarettes per day, insomnia, and sedentary behavior were identified as risk factors for shoulder periarthritis. Based on these modifiable risk factors, a VR exercise intervention system was designed.

The VR Harvest intervention within the system is designed to directly target shoulder muscles, aiding in the improvement of shoulder joint function in patients with shoulder periarthritis through a series of shoulder-strengthening exercises. This approach aims to alleviate pain, increase shoulder mobility, and enhance daily functional capacity. Additionally, the training intensity can be adjusted to increase exercise volume, which helps reduce BMI and lower the risk of developing shoulder periarthritis. The VR Harvest intervention developed in this study is as effective as traditional exercise therapy for treating shoulder pain. By gamifying the exercise process, VR Harvest enhances the enjoyment and engagement of workouts, making it easier for users to adhere to the program.

However, this study has several limitations. First, while MR analysis provides advantages in inferring causal relationships, the complexity of genetic variation makes it challenging to fully exclude the influence of pleiotropy. This may introduce some bias in our understanding of the causal relationships between certain factors and shoulder periarthritis. Second, the sample in this study primarily consists of participants from a specific region, which limits its representativeness. Consequently, the findings may not be directly generalizable to all populations. Differences in lifestyle, genetic background, and other factors across regions and ethnicities may affect the occurrence and progression of shoulder periarthritis. Additionally, the long-term effects of the VR exercise intervention system require further follow-up and investigation. While we observed positive short-term effects in improving shoulder periarthritis symptoms and reducing BMI, it remains to be seen whether these effects are sustainable over time and whether other potential issues may arise, necessitating longer-term observation and analysis.

Looking ahead, we aim to further expand and deepen this research. We plan to increase the sample size to include participants from a broader range of ages, genders, and regions, thereby enhancing the generalizability and applicability of our findings. By diversifying the sample, we can gain a more comprehensive understanding of the mechanisms and characteristics of shoulder periarthritis across different populations, providing a stronger foundation for developing more effective prevention and treatment strategies. Additionally, we will continue to optimize the VR exercise intervention system by creating more personalized training programs tailored to the specific needs and conditions of individual patients. For instance, we will offer varying intensity and difficulty levels for patients with different degrees of severity and design exercise modes and virtual environments suitable for various ages and physical conditions. Furthermore, we plan to integrate artificial intelligence and big data technologies to more accurately monitor and assess patients' rehabilitation progress. By collecting and analyzing data from the training process—such as movement posture, exercise intensity, and pain levels—we can adjust treatment plans in real time to enhance rehabilitation outcomes. Moreover, utilizing big data analytics, we can analyze large-scale patient rehabilitation data to uncover patterns and trends, providing more robust scientific evidence for the treatment of shoulder periarthritis.

## Figures and Tables

**Table 1:** IVs for BMI, cigarettes per day, insomnia, sedentary behavior, and educational attainment.

SNP	EA	OA	EAF	Beta	Se	*p*
BMI						
rs1804528	A	G	0.351	0.0109	0.0020	3.0 × 10^−8^
rs2423668	C	T	0.551	−0.0105	0.0019	2.8 × 10^−8^
Cigarettes per day						
rs2084533	T	C	0.319	0.0166	0.0029	1.22 × 10^−8^
rs215600	A	G	0.640	−0.0246	0.0029	1.1 × 10^−17^
Insomnia						
rs11097861	A	G	0.284	−0.0091	0.0015	1.7 × 10^−9^
rs11184946	C	T	0.582	−0.0087	0.0014	2.9 × 10^−10^
Sedentary behavior						
rs10041724	T	C	0.807	0.0180	0.0027	3.9 × 10^−11^
rs10054327	G	A	0.576	0.0172	0.0021	3.4 × 10^−15^
rs10145592	C	G	0.409	−0.0148	0.0022	1.8 × 10^−11^
Educational attainment						
rs10760199	T	G	0.551	0.0115	0.0020	2.5 × 10^−8^
rs114408770	A	G	0.024	−0.0436	0.0068	1.6 × 10^−10^

*Note:* beta, effect size.

Abbreviations: BMI, body mass index; EA, effect allele; EAF, effect allele frequency; IVs, instrumental variables; OA, other allele; SE, standard error; SNP, single nucleotide polymorphism.

**Table 2:** Results of the statistical analyses of the associations between exposure factors and shoulder periarthritis.

Exposure	Methods	SNP	Beta	Se	*p*
BMI	IVW	450	0.2061	0.0370	2.61 × 10^−8^
	MR–Egger regression	450	0.0896	0.1075	0.4047
	WM	450	0.1425	0.0493	0.0038
	Weighted mode	450	0.0764	0.1239	0.5375

Sedentary behavior	IVW	129	0.3360	0.0652	2.59 × 10^−7^
	MR–Egger regression	129	0.4734	0.3019	0.1193
	WM	129	0.2051	0.0835	0.0142
	Weighted mode	129	0.2392	0.2392	0.7780

Cigarettes per day	IVW	18	0.2464	0.1015	0.0152
	MR–Egger regression	18	0.1385	0.2307	0.5564
	WM	18	0.2215	0.1344	0.0993
	Weighted mode	18	0.2035	0.1429	0.1723

Insomnia	IVW	29	0.8681	0.2579	0.0007
	MR–Egger regression	29	0.8412	0.7203	0.2530
	WM	29	0.7586	0.2870	0.0082
	Weighted mode	29	0.7187	0.3844	0.0720

Educational attainment	IVW	32	−0.8595	0.1649	1.89 × 10^−7^
	MR–Egger regression	32	−0.5558	0.6440	0.3950
	WM	32	−0.6691	0.2022	0.0009
	Weighted mode	32	−0.3623	0.4703	0.4468

Abbreviations: BMI, body mass index; IVW, inverse variance weighted; MR, Mendelian randomization; SNP, single nucleotide polymorphism; WM, weighted median.

**Table 3:** MR sensitivity analysis results for shoulder periarthritis.

Exposure	Outcome	Cochran's tests for heterogeneity				Horizontal pleiotropy		
		MR-Egger *Q*	MR-Egger *p*	IVW Q	IVW *p*	MR-Egger intercept	MR-Egger Se	MR-Egger *p*
BMI	Shoulder periarthritis	690.1502	<0.01	692.2013	<0.01	0.0019	0.0016	0.2491
Cigarettes per day	Shoulder periarthritis	20.6584	0.1920	21.0119	0.2257	0.0032	0.0062	0.6079
Sedentary behavior	Shoulder periarthritis	182.6326	0.0009	182.9451	0.0010	−0.0023	0.0048	0.6149
Educational attainment	Shoulder periarthritis	58.3189	0.0014	58.7825	0.0018	−0.0047	0.0096	0.6288
Insomnia	Shoulder periarthritis	60.1694	0.0002	60.1730	0.0003	0.0003	0.0074	0.9683

Abbreviations: BMI, body mass index; IVW, inverse variance weighted; MR, Mendelian randomization.

**Table 4:** Experimental movement schemes of VR exercise therapy and traditional exercise therapy.

Experimental group (VR exercise)	Control group (traditional exercise)
Pulley pull exercise: Install a pulley on a wall or tree and pass a rope through it. Tie a small wooden stick at each end. Pull up and down for exercise.	Lateral raise stretch: Hold dumbbells or water bottles with both hands. Keep the arms straight and slowly lift them from both sides of the body to the horizontal position, then slowly lower them.
Wall climbing exercise: Picking an apple, place the apple at the highest point.	Front raise stretch: Hold dumbbells or water bottles with both hands. Keep the arms straight and slowly lift them from the front of the body to the horizontal position, then slowly lower them.
Wing stretch exercise: Stand up. After getting the apple, let the upper limbs hang down naturally. Straighten the arms and slowly raise the arms upward. Put the apple into the boxes on both sides at the same time and hold the movement for 10 s.	Bent-over fly stretch: Stand with feet shoulder-width apart and slightly bend the knees. Lean forward slightly. Hold dumbbells or water bottles with both hands and let the arms hang down naturally. Then lift the arms to the sides until they are parallel to the ground.
Head brushing exercise: Alternately touch the apple with hands in turn from the forehead, the top of the head, the back of the head, and behind the ears.	Lucky cat stretch: Hold a dumbbell in each hand. Bend the elbow at 90°. Lift the arm upward so that the upper arm is parallel to the ground and the forearm is perpendicular to the upper arm. Then, with the shoulder as the axis, rotate the forearm upward until the forearm is vertically upward. Then slowly return to the starting position.
Elbow flexing and arm swinging exercise: Keep the upper arm close to the body. Bend the elbow. After getting the apple, use the elbow joint as the fulcrum to do external rotation. Put the apple into the baskets on both sides.	Shoulder circling stretch: Let the hands hang down naturally. Slowly circle around with the shoulder as the center, from front to back first, and then from back to front.

Abbreviation: VR, virtual reality.

**Table 5:** Changes in BMI values of subjects with similar initial BMI values after 4 weeks of exercise intervention.

Group	Initial	End	*Δ*BMI
Control group	27.44	27.25	0.00706
	24.69	24.48	0.00860
	22.42	22.31	0.00478
	20.68	20.61	0.00332
	23.53	23.40	0.00538

Experimental group	27.46	26.92	0.01957
	24.69	24.29	0.01620
	22.45	22.23	0.00979
	20.66	20.46	0.00978
	23.28	23.02	0.01136

Abbreviation: BMI, body mass index.

**Table 6:** Wilcoxon signed-rank test comparison of pre- and postintervention VAS scores and BMI changes within groups.

Group	Variable	*N*	Positive ranks	Negative ranks	Ties	*Z*	*p*-Value
Experimental group	The difference in VAS score	5	0	5	0	−2.236	0.025
	The difference in BMI	5	0	5	0	−2.023	0.043

Control group	The difference in VAS score	5	0	2	3	−1.414	0.157
	The difference in BMI	5	0	5	0	−2.023	0.043

Abbreviations: BMI, body mass index; VAS, visual analog scale.

**Table 7:** Mann–Whitney *U* test comparing postintervention VAS scores and BMI changes between experimental and control groups.

Variable	*N* (Total sample size)	*U* value	Wilcoxon *W* value	*Z* value	Standard error	Asymptotic significance (two-sided)	Exact significance (two-sided)
Differences in VAS scores	10	14.500	29.500	0.430	4.655	0.667	0.690
*Δ*BMI	10	0.000	15.000	0.000	4.787	0.009	0.008

Abbreviations: BMI, body mass index; VAS, visual analog scale.

## Data Availability

The data sets used and analyzed during the current study are available from IEU Open GWAS project (ieu-b-40, ukb-b-6134), GWAS Catalog (https://www.ebi.ac.uk/gwas/publications/32317632, https://www.ebi.ac.uk/gwas/publications/30643251 and https://ftp.ebi.ac.uk/pub/databases/gwas/summary_statistics/GCST007001-GCST008000/GCST007387/), and FinnGen (https://r8.finngen.fi/pheno/M13_SHOULDERNAS).

## Supporting Information

Supporting Information Table S1: Changes in BMI values of subjects after 4 weeks of exercise intervention
