# Supplementary material for: Pathogenic Factor Analysis of Shoulder Periarthritis and Design of Virtual Reality Exercise Intervention System
Source: Appl Bionics Biomech. 2024 Dec 19;2024:6543337. doi: 10.1155/abb/6543337 (PMC11671636; doi:10.1155/abb/6543337)
Supplement: Supporting Information — Table S1: Changes in BMI values of subjects after 4 weeks of exercise intervention [file 6543337.f1.doc]

**Supplementary Information**

**Pathogenic Factor Analysis of Shoulder Periarthritis and Design of Virtual Reality Exercise Intervention System**

Yucheng Tian^1^, Dan Qiu^1^, Renjie Song^1^，Xue Cheng^1^, Feiyu Chen^1^, Dongqing Sun^1^, Yiduo Zhou^1^ ，Shaomin Cai^4^, Zhaowei Wang^4^, Weijia Zhang^1，2，3*^

^1^ School of Mathematical and Information Science, Shaoxing University, Shaoxing, 312000, China.

^2^ Key Laboratory of Artificial Intelligence Applications, Shaoxing University, Shaoxing, China

^3^ Visiting Scholar, Department of AOP Physics, University of Oxford, Oxford, UK

^4^ School of Medicine, Shaoxing University, Shaoxing, China

Table s1. Changes in BMI values of subjects after four weeks of exercise intervention

|  | Initial | First week | Second week | Third week | The fourth week | ΔBMI |
| --- | --- | --- | --- | --- | --- | --- |
| Control group | 27.44 | 27.38 | 27.33 | 27.32 | 27.25 | 0.00706 |
|  | 24.69 | 24.67 | 24.61 | 24.53 | 24.48 | 0.00860 |
|  | 22.42 | 22.41 | 22.39 | 22.37 | 22.31 | 0.00478 |
|  | 20.68 | 20.67 | 20.65 | 20.62 | 20.61 | 0.00332 |
|  | 23.53 | 23.50 | 23.48 | 23.45 | 23.40 | 0.00538 |
| Experimental group | 27.46 | 27.33 | 27.18 | 27.06 | 26.92 | 0.01957 |
|  | 24.69 | 24.63 | 24.53 | 24.38 | 24.29 | 0.01620 |
|  | 22.45 | 22.40 | 22.34 | 22.25 | 22.23 | 0.00979 |
|  | 20.66 | 20.63 | 20.59 | 20.50 | 20.46 | 0.00978 |
|  | 23.28 | 23.23 | 23.16 | 23.08 | 23.02 | 0.01136 |
